# NGF sensitizes TrkA SH-SY5Y neuroblastoma cells to TRAIL-induced apoptosis

**DOI:** 10.1038/cddiscovery.2016.4

**Published:** 2016-02-01

**Authors:** P Ruggeri, L Cappabianca, A R Farina, L Gneo, A R Mackay

**Affiliations:** 1 Department of Applied Clinical and Biotechnological Sciences, University of L'Aquila, Via Vetoio, Coppito 2, L’Aquila 67100, Italy

## Abstract

We report a novel pro-apoptotic function for nerve growth factor (NGF) and its tropomyosin-related kinase A (TrkA) receptor in sensitizing TRAIL (TNF-related apoptotis-inducing ligand)-resistant SH-SY5Y neuroblastoma (NB) cells to TRAIL-induced apoptosis, resulting in the abrogation of anchorage-independent tumourigenic growth *in vitro*. We show that the TRAIL-resistant SH-SY5Y phenotype is cFLIP (cellular FLICE-like inhibitory protein) dependent and not due to low-level functional TRAIL receptor or caspase expression or an inhibitory equilibrium between functional and decoy TRAIL receptors or B-cell lymphoma 2 (Bcl-2) and BH3-only (Bcl-2 homology domain 3-only) family proteins. NGF sensitization of SH-SY5Y cells to TRAIL-induced apoptosis was dependent upon TrkA expression, activation and subsequent sequestration of cFLIP. This reduces cFLIP recruitment to TRAIL-activated death receptors and increases the recruitment of caspase-8, leading to TRAIL-induced, caspase-dependent, type II apoptosis via the intrinsic mitochondrial pathway. This effect was temporary, inhibited within 6 h by nuclear factor-*κ* binding (NF-*κ*B)-mediated increase in myeloid cell leukaemia-1 (Mcl-1) expression, abrogated by transient cFLIP or B-cell lymphoma-extra large (Bcl-xL) overexpression and optimized by NF-*κ*B and Mcl-1 inhibitors. This novel mechanism adds an important pro-apoptotic immunological dimension to NGF/TrkA interaction that may not only help to explain the association between TrkA expression, better prognosis and spontaneous remission in NB, but also provides a novel potential pro-apoptotic therapeutic use for NGF, TRAIL and inhibitors of NF-*κ*B and/or Mcl-1 in favourable and unfavourable NBs that express TrkA and exhibit cFLIP-mediated TRAIL resistance.

## Introduction

During sympathetic nervous system (SNS) development, neuroblasts delaminate from the neural crest and migrate to form para-aortic sympathetic ganglia before differentiating into the various cellular components of the SNS. This finely tuned process is associated with both resistance and sensitivity to apoptosis, ensuring sufficient cell numbers to form the fully functional SNS, while eliminating cellular excess. Within this context, neuroblast resistance and sensitivity to apoptosis is regulated by neurotrophins (nerve growth factor (NGF), BDNF, NT3 and NT4/5) and their corresponding receptors (tropomyosin-related kinase (Trk)-A, TrkB, TrkC, p75^NTR^ and Sortilin). Active neurotrophins ligate TrkA, TrkB and TrkC to protect neuroblasts from apoptosis; TrkC in the absence of p75^NTR^ and TrkA in the presence of p75^NTR^ are pro-apoptotic in the absence of active neurotrophins, whereas p75^NTR^ and Sortilin are pro-apoptotic upon pro-neurotrophin ligation in the absence of Trk receptor expression. This variety of potential pro- and anti-apoptotic outcomes is regulated by spatial and temporal changes in neurotrophin and neurotrophin receptor expression.^1^

Apoptosis is activated via the extrinsic and/or intrinsic pathways.^2,3^ The identification of tumour necrosis factor-*α* (TNF*α*) as the pro-apoptotic cytokine responsible for endotoxin-induced tumour necrosis^4^ paved the way for characterization of the extrinsic pathway and the subsequent identification of the TNF*α* pro-apoptotic cytokine family comprising TNF*α*, Apo1/Fas, Apo2L/TRAIL (TNF-related apoptotis-inducing ligand) and Apo3L that induce two types of apoptotic response through their cognate TNFR1, Apo1/FasL, FasR, Apo3L/DR3, Apo2L/DR4 and DR5 death receptors.^5,6^ In type I cells, activated death receptors recruit FADD (Fas-associated protein with death domain) and caspase-8 (or caspase-10) to induce apoptosis via the activation of caspase-7 and caspase-3. In type II cells, low-level caspase-8 activation does not result in the direct activation of caspase-3 but cleaves the pro-apoptotic BH3-only (Bcl-2 homology domain 3-only) protein BID (BH3 interacting domain death agonist). Cleaved tBID translocates to the mitochondria and induces permeabilization of the outer mitochondrial membrane (OMM). This results in the release of the pro-apoptotic mitochondrial proteins cytochrome *C*, Omi/Htr2a and SMAC/DIABLO (second mitochondria-derived activator of caspases) into the cytosol that results in caspase-3-dependent apoptosis^3,7^ and/or caspase-independent death through the release of mitochondrial apoptosis-inducing factors (AIFs) and endonuclease G.^8^

Apo2L/TRAIL is a promising chemotherapeutic agent because of its selective pro-apoptotic action on tumour but not nontransformed cells^9–12^ that exhibit a multiplicity of redundant TRAIL resistance pathways.^13^ In tumour cells, TRAIL induces apoptosis by ligating functional DR4 and/or DR5 TRAIL receptors that recruit FADD and initiator caspase-8 (or -10) to the death-inducing complex DISC.^5,6,14^ In both type I and type 2 tumour cells, TRAIL-induced apoptosis is regulated by the equilibrium between functional (DR4 and DR5) and decoy (DcR1, DcR2 and OPG) TRAIL receptors, the level of caspase-3, caspase-8, caspase-9 and caspase-10 expression and the equilibrium between caspase-8 and its inhibitory analogues cFLIP-S and cFLIP-L. In type 2 but not type I cells, TRAIL-induced apoptosis is also regulated by the equilibrium between pro-apoptotic BH3-only and anti-apoptotic B-cell lymphoma 2 (Bcl-2) family proteins that regulate OMM permeability.^2,3,5–9,15^

Neuroblastoma (NB) originates from neural crest-derived neuroblasts transformed at different stages along the sympathoadrenal lineage, blocked in differentiation and selected for resistance to apoptosis by a combination of oncogene activation, oncosuppressor inactivation and physiological apoptosis protection mechanism(s) conserved at the time of transformation.^1^ The characterization of these mechanisms is critical for developing novel pro-apoptotic therapeutic strategies.

The therapeutic application of TRAIL in NB has been hampered by reports of TRAIL resistance in NB cell lines and models,^16–30^ attributed to methylation-mediated caspase-8 silencing, altered expression of decoy and functional TRAIL receptors, high-level expression of cFLIP-S and/or cFLIP-L and protective IP3K/Akt/mTor and/or RIP/NF-*κ*B signalling that promote an anti-apoptotic equilibrium between pro-apoptotic BH3-only and anti-apoptotic Bcl-2 family proteins.^19–30^ Sensitization of NB cells to TRAIL-induced apoptosis has been reported for demethylating agents that increase caspase-8 expression; DR5 TRAIL receptor transduction, cFLIP (cellular FLICE-like inhibitory protein) and nuclear factor-*κ* binding (NF-*κ*B) inhibitors and drugs that promote a pro-apoptotic patterns of TRAIL-induced apoptosis component expression.^18,20,28,29,31–40^

In the present study, we characterize a novel mechanism for sensitizing SH-SY5Y NB cells to TRAIL-induced apoptosis that involves NGF and its receptor TrkA. This novel mechanism not only adds a pro-apoptotic immunological dimension to NGF/TrkA interaction in neuroblasts but also provides a novel potential pro-apoptotic therapeutic strategy for the use of NGF and TRAIL in TrkA-expressing NB.

## Results

### NGF sensitizes TRAIL-resistant TrkA SH-SY5Y cells to TRAIL-induced apoptosis

**Figure 1:**
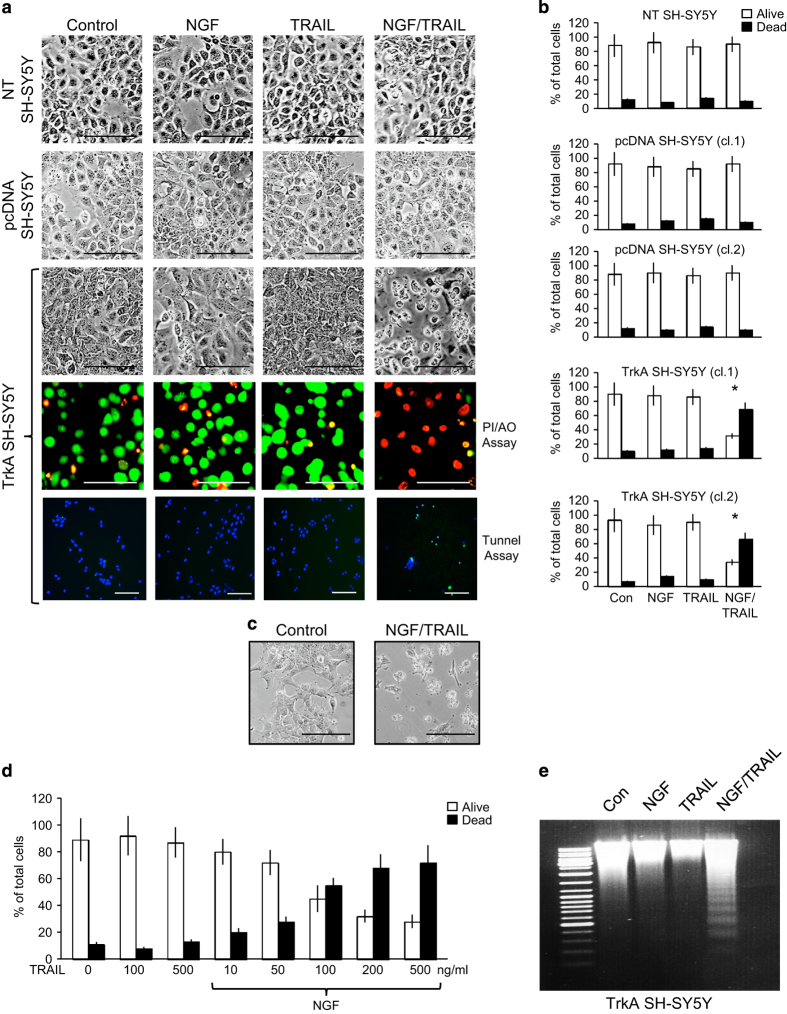
NGF sensitizes TrkA SH-SY5Y cells to TRAIL-induced apoptosis. (**a**) Representative phase contrast and fluorescent micrographs (bar=100 *μ*M) and (**b**) histograms demonstrating the marked induction of TrkA SH-SY5Y but not NT or pcDNA SH-SY5Y cell death following 16 h of incubation with NGF (100 ng/ml) plus TRAIL (200 ng/ml), compared with the lack of cell death in all three cell lines following overnight (16 h) incubation with either NGF (100 ng/ml) or TRAIL (200 ng/ml) alone. Results are displayed as the mean percentage (%) of alive (white) and dead (black) cells (±S.D.) in three independent experiments each performed in duplicate (*statistical significance). (**c**) Representative phase contrast micrographs demonstrating the effect of NGF plus TRAIL on the morphology of surviving TrkA SH-SY5Y cells. (**d**) Histogram demonstrating dose-dependent induction of TrkA SH-SY5Y cell death by TRAIL (10–500 ng/ml) in the presence but not absence of NGF (100 ng/m). Results are displayed as the mean percentage (%) (±S.D.) of alive (white) and dead (black) cells in three independent experiments each performed in duplicate. (**e**) Representative ethidium bromide stained agarose gel demonstrating laddering of DNA purified from TrkA SH-SY5Y cells treated for 16 h with NGF (100 ng/ml) plus TRAIL (200 ng/ml) but not from TrkA SH-SY5Y cells treated with either NGF (100 ng/ml) or TRAIL (200 ng/ml) alone.

In two-dimensional culture, TRAIL at concentrations from 10 ng to 1 *μ*g/ml did not induce significant death of either nontransfected (NT), duplicate empty pcDNA3.1 vector (pcDNA)-transfected or duplicate TrkA-transfected SH-SY5Y cell lines, confirming their TRAIL-resistant phenotypes (Figures 1a and b). NGF (100 ng/ml) also failed to induce significant death of NT SH-SY5Y, pcDNA SH-SY5Y or TrkA SH-SY5Y cells (Figures 1a and b). In contrast, the combination of NGF (100 ng/ml) and TRAIL (200 ng/ml) induced a mean (±S.D.) of 68.7±13.8 and 66±14.3% death in the two TrkA SH-SY5Y cell lines but did not induce the death of either NT SH-SY5Y or pcDNA SH-SY5Y cells (Figures 1a and b). TrkA SH-SY5Y cells that survived the effects of NGF plus TRAIL exhibited neurite-like projections, suggesting the initiation of neural differentiation (Figure 1c). In concentration studies, the death of TrkA SH-SY5Y cells in the presence of NGF (100 ng/ml) was detected at TRAIL concentrations ≥50 ng/ml and plateaued with a mean (±S.D.) of 68.3±15.3% death at a concentration of 200 ng/ml (Figures 1a and d). The TRAIL-induced death of TrkA SH-SY5Y cells was confirmed to be apoptotic by Tunel assay (Figure 1a), detection of DNA laddering (Figure 1e) and was abrogated by the caspase inhibitors z-DEVD-fmk (caspase-3), z-IETD-fmk (caspase-8) and z-LEHD-fmk (caspase-9) (see Figures 4d and e).

### NGF/TRAIL inhibits TrkA SH-SY5Y tumourigenic growth *in vitro*

The effect of TRAIL and NGF upon the tumourigenic activity of NT, pcDNA and TrkA SH-SY5Y cell lines was assessed in substrate-independent tumourigenesis assays *in vitro*.

**Figure 2:**
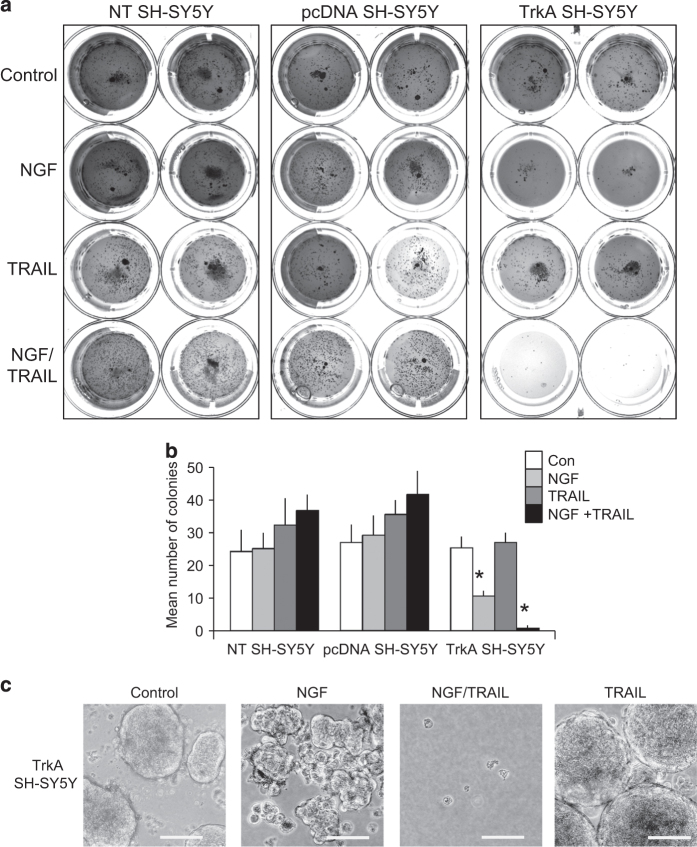
NGF plus TRAIL abrogates the tumourigenic activity of TrkA SH-SY5Y cells *in vitro*. (**a**) Representative photograph and (**b**) histogram demonstrating significant inhibition of TrkA SH-SY5Y but not NT SH-SY5Y or pcDNA SH-SY5Y tumourigenic activity *in vitro* by NGF (100 ng) alone or in combination with TRAIL (200 ng). Results are displayed as the mean (±S.D.) number of tumours in 10×10 magnification fields in triplicate experiments each performed in duplicate (*statistical significance). (**c**) Representative phase contrast micrographs demonstrating tumour spheroid appearance of TrkA SH-SY5Y cells *in vitro* in the absence (control) or presence of NGF (100 ng/ml), TRAIL (200 ng/ml) plus NGF (100 ng/ml) or TRAIL (200 ng/ml) alone.

NT, pcDNA and TrkA SH-SY5Y cells formed similar numbers of similar-sized tumour spheroids over 14 days in soft agar tumourigenesis assays, under basal conditions (Figures 2a and b, identical results were obtained for duplicate cell lines and results are displayed for single NT, pcDNA and TrkA SH-SY5Y cell lines). The addition of TRAIL (200 ng/ml) to soft agar did not significantly reduce the tumourigenic activity of NT SH-SY5Y, pcDNA SH-SY5Y or TrkA SH-SY5Y cells (Figures 2a and b). NGF (100 ng/ml) added to soft agar significantly reduced the number of tumour spheroids formed by TrkA SH-SY5Y cells by a mean (±S.D.) of 58.5±9.1% (*P*<0.0001, d.f.=10) but did not inhibit the number of tumour spheroids formed by either NT SH-SY5Y or pcDNA SH-SY5Y cells over 14 days (Figures 2a and b). Furthermore, tumour spheroids formed by TrkA SH-SY5Y cells in the presence of NGF (100 ng/ml) appeared to be less well formed (Figure 2c). The addition of NGF (100 ng/ml) plus TRAIL (200 ng/ml) to soft agar abrogated tumour spheroid growth by TrkA SH-SY5Y cells, resulting in a mean (±S.D.) of 96.8% (*P*<0.0001, d.f.=10) inhibition when compared with untreated TrkA SH-SY5Y cells and 92.5% (*P*<0.0001, d.f.=10) inhibition when compared with NGF-treated TrkA SH-SY5Y cells (Figures 2a and b). In contrast, the combination of NGF plus TRAIL did not reduce the number of tumour spheroids formed by NT SH-SY5Y or pcDNA SH-SY5Y cells (Figures 2a and b).

### SH-SY5Y cells express all components required for TRAIL-induced apoptosis

**Figure 3:**
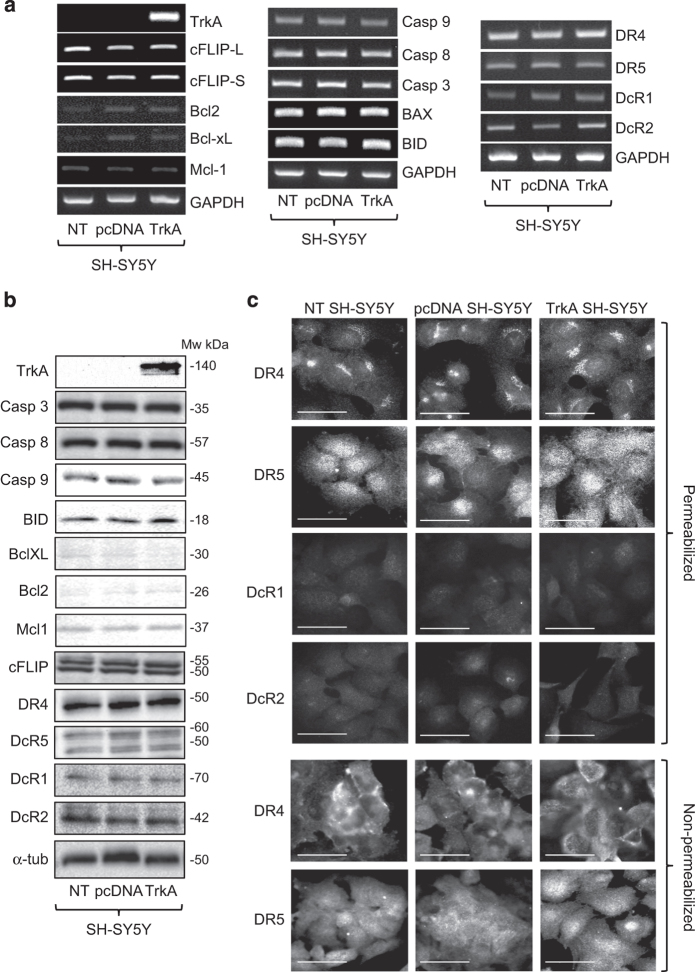
SH-SY5Y cells express all of the major components required for TRAIL-induced apoptosis. (**a**) Ethidium bromide-stained agarose gels and (**b**) western blots demonstrating similar RT-PCR product and protein levels for all of the major components involved in TRAIL-induced apoptosis in NT-SH-SY5Y, pcDNA SH-SY5Y and TrkA SH-SY5Y cells. (**c**) Representative immunofluorescent micrographs demonstrating close similarity in the expression of functional DR4 and DR5 and DcR1 and DcR2 TRAIL receptors in permeabilized cells and similar levels of cell surface DR4 and DR5 immunoreactivity in nonpermeabilized NT-SH-SY5Y, pcDNA SH-SY5Y and TrkA SH-SY5Y cells (bar=50 *μ*M).

**Figure 4:**
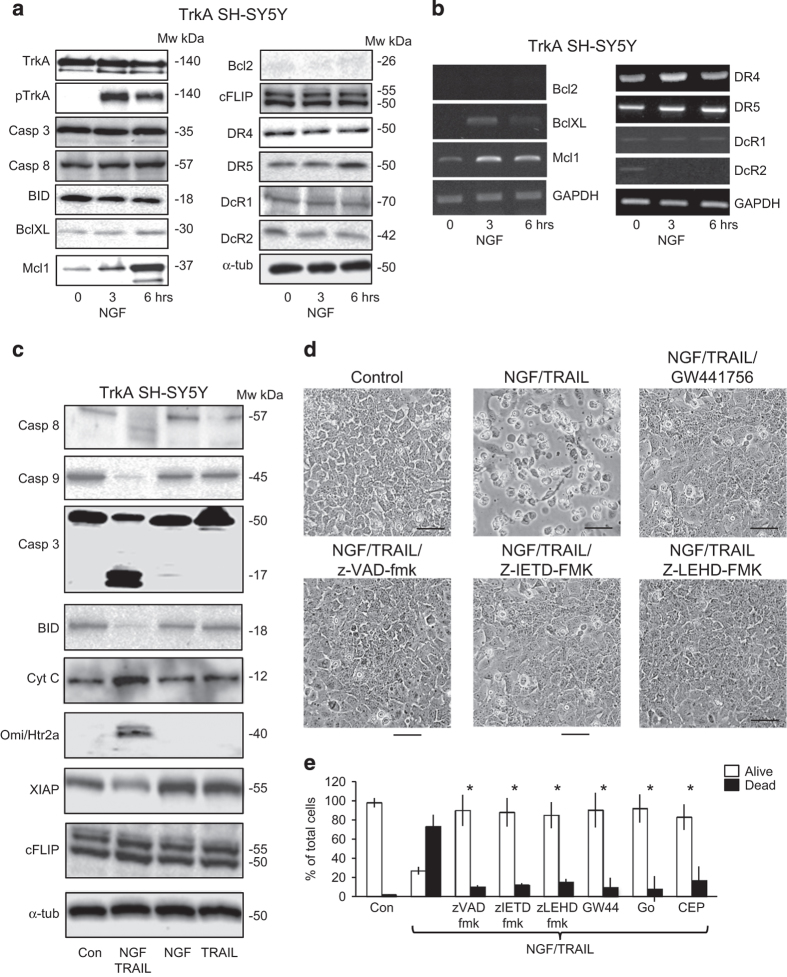
NGF sensitizes TrkA SH-SY5Y cells to TRAIL-induced caspase-dependent type II apoptosis and regulates Mcl-1 but not FLIP expression. (**a**) Western blots demonstrating NGF stimulation of Mcl-1, Bcl-xL and DR5 protein expression, and the maintenance of TrkA tyrosine phosphorylation in TrkA SH-SY5Y cells over a 1 to 6 h time course. (**b**) Agarose gels demonstrating NGF stimulation of Mcl1 and Bcl-xL but not Bcl-2 mRNA expression in TrkA SH-SY5Y cells over a 1–6 h time course. (**c**) Representative western blots demonstrating changes in cytoplasmic caspase-8, caspase-9, caspase-3, BID, cytochrome *C*, OMI/Htr2a and XIAP but not in cFLIP or *α*-tubulin in TrkA SH-SY5Y cells treated with a combination of NGF (100 ng/ml) plus TRAIL (200 ng/ml) but not with NGF (100 ng/ml) or TRAIL (200 ng/ml) alone. (**d**) Representative phase contrast micrographs (bar=100 *μ*M) and (**e**) histogram demonstrating the inhibition of TRAIL-induced apoptosis by z-VAD-fmk, z-IETD-fmk, z-LEHD-fmk caspase inhibitors and the TrkA kinase inhibitors CEP-701 (CEP), Gö6976 (Go) and GW441756 (GW) (histogram only). Results are displayed as the mean percentage (%) (±S.D.) alive (white) or dead (black) cells in three experiments, each performed in duplicate (*statistical significance).

Molecular characterization of the TRAIL-resistant SH-SY5Y phenotype revealed similar levels of constitutive mRNA and protein expression for functional (DR4 and DR5) and decoy (DcR1 and DcR2) TRAIL receptors; caspase-3, caspase-8 and caspase-9; cFLIP-S and cFLIP-L; Bcl-2, B-cell lymphoma-extra large (Bcl-xL), myeloid cell leukaemia-1 (Mcl-I) and BID in NT SH-SY5Y, pcDNA SH-SY5Y and TrkA SH-SY5Y cell lines (Figures 3a and b, identical results were obtained for duplicate cell lines and results are displayed for single NT, pcDNA and TrkA SH-SY5Y cell lines). Indirect IF confirmed similar levels of DR4, DR5, DcR1 and DcR2 TRAIL receptors in NT SH-SY5Y, pcDNA SH-SY5Y and TrkA SH-SY5Y cells, with an equilibrium in favour of functional rather than decoy receptors. Indirect IF confirmed cell surface DR4 and DR5 receptor expression in nonpermeabilized cells (Figure 3c). In TrkA SH-SY5Y cells, NGF (100 ng/ml for 3 and 6 h) alone increased the expression of Mcl-1, Bcl-xL and DR5, decreased DcR2 expression, did not alter the expression of DR4, DcR1, caspase-3, caspase-8, caspase-9, cFLIP-S, cFLIP-L, Bcl-2 or BID and did not regulate the expression of any of these genes in either NT SH-SY5Y or pcDNA SH-SY5Y cells (Figures 4a and b, data displayed for TrkA SH-SY5Y cells only). TRAIL (200 ng/ml for 3 and 6 h) alone did not alter the expression of these genes in NT SH-SY5Y, pcDNA SH-SY5Y or TrkA SH-SY5Y cells (data not displayed). These data indicate that the TRAIL-resistant SH-SY5Y phenotype does not result from a lack of expression of any single component of TRAIL-induced apoptosis; TrkA *per se* does not alter the expression of components of TRAIL-induced apoptosis in SH-SY5Y cells and NGF stimulates Mcl-1 and to a lesser extent Bcl-xL, DR4 and DR5 expression in TrkA SH-SY5Y cells.

### NGF plus TRAIL induce type II apoptosis in TrkA SH-SY5Y cells

TRAIL-induced apoptosis of NGF-sensitized TrkA SH-SY5Y cells was associated with the cleavage of BID, caspase-3, caspase-8 and caspase-9, increased cytosolic levels of the mitochondrial proteins cytochrome *C* and Htr2a/OMI and reduced cytosolic levels of the caspase inhibitor XIAP (X-linked inhibitor of apoptosis) (Figure 4c, results displayed for a single TrkA SH-SY5Y cell line only). The caspase-3 inhibitor z-DEVD-fmk (100 *μ*M), caspase-8 inhibitor z-IETD-fmk (20 *μ*M) and the capase-9 inhibitor z-LEHD (50 *μ*M) all significantly inhibited TRAIL-induced apoptosis of NGF-sensitized TrkA SH-SY5Y cells (Figure 4d) from a mean (±S.D.) of 73.2±17% in untreated controls to 10±18% (*P*<0.0001, d.f.=10), 12±16.3% (*P*<0.0001, d.f.=10) and 15±23% (*P*=0.0005, d.f.=10), respectively (Figure 4e). The TrkA tyrosine kinase inhibitors CEP-701,^41^ Gö6976^42^ and GW441756^43^ also significantly inhibited TRAIL-induced apoptosis of NGF-sensitized TrkA SH-SY5Y cells from a mean (±S.D.) of 73.2±17% in controls to 17±14.5% (*P*<0.0001, d.f.=10), 8±13.3% (*P*<0.0001, d.f.=10) and 9.8±10% (*P*<0.0001, d.f.=10), respectively (Figure 4d, phase contrast GW441756 only; and Figure 4e).

**Figure 5:**
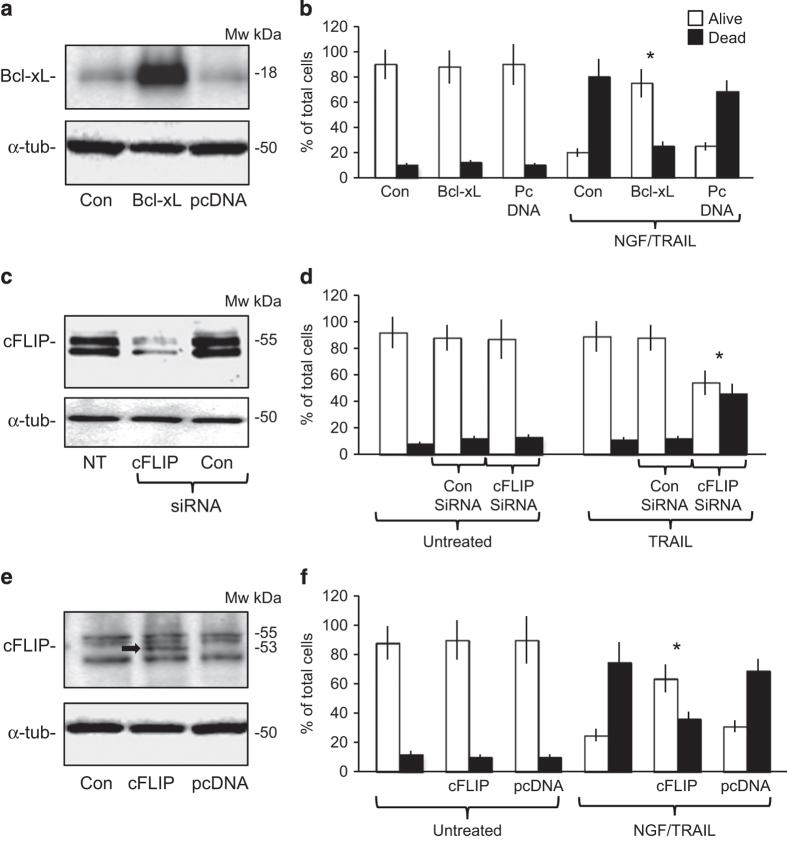
Bcl-xL and cFLIP expression block TRAIL-induced apoptosis in NGF-sensitized TrkA SH-SY5Y cells and cFLIP is responsible for the TRAIL-resistant SH-SY5Y phenotype. (**a**) Western blot demonstrating transient Bcl-xL overexpression in TrkA SH-SY5Y cells transiently transfected with a mammalian pcDNA3.1 Bcl-xL expression vector but not with empty pcDNA3.1 vector. (**b**) Histogram demonstrating significant (*) inhibition of TRAIL-induced apoptosis in NGF-treated Bcl-xL-transduced but not empty vector-transfected TrkA SH-SY5Y cells in response to NGF (100 ng/ml) plus TRAIL (200 ng/ml). (**c**) Representative western blots demonstrating cFLIP-specific siRNA but not control siRNA knockdown of cFLIP but not *α*-tubulin in TrkA SH-SY5Y cells. (**d**) Histogram demonstrating a significant (*) increase in apoptosis of TrkA SH-SY5Y cells exhibiting siRNA cFLIP knockdown (cFLIP siRNA) but not in cells transiently transfected with control siRNA duplexes (Con SiRNA) in the presence but not absence of TRAIL and in the absence of NGF. (**e**) Representative western blot demonstrating transient overexpression of FLAG-tagged cFLIP in TrkA SH-SY5Y cells but not in cells transfected with empty pcDNA expression vector. (**f**) Histogram demonstrating a significant (*) reduction in TrkA SH-SY5Y apoptosis induced by NGF (100 ng/ml) plus TRAIL (200 ng/ml) in TrkA SH-SY5Y cells that transiently overexpress exogenous FLAG-tagged cFLIP compared with empty expression vector-transfected counterparts. Histogram results are displayed as the mean percentage (%) (±S.D.) alive (white) or dead (black) cells in three experiments, each performed in duplicate (*statistical significance).

Transient Bcl-xL overexpression (Figure 5a) significantly reduced TRAIL-induced apoptosis in NGF-sensitized TrkA SH-SY5Y cells from a mean (±S.D.) of 68.5±13 to 25.1±15% (*P*<0.0003, d.f.=10; Figure 5b). These data confirm that TRAIL-induced apoptosis of NGF-sensitized TrkA SH-SY5Y cells is dependent upon TrkA activity, is caspase dependent and is mediated through the intrinsic mitochondrial pathway.

### The TRAIL-resistant SH-SY5Y phenotype is cFLIP dependent

NT SH-SY5Y, pcDNA SH-SY5Y and TrkA SH-SY5Y cells all exhibited similar constitutive high-level cFLIP mRNA and protein expression (Figures 3a and b). The small interfering RNA (siRNA) cFLIP knockdown (Figure 5c) sensitized TrkA SH-SY5Y cells to TRAIL-induced apoptosis in the absence of NGF, resulting in a mean (±S.D.) of 46±16.2% death (*P*<0.0001, d.f.=10, compared with siRNA-transfected controls; Figure 5d). Transient cFLIP overexpression (Figure 5e) significantly reduced TRAIL-induced apoptosis in NGF-sensitized TrkA SH-SY5Y cells from a mean (±S.D.) of 69±11.6% to 36.4±12.6% (*P*=0.0009, d.f.=10; Figure 5f). These data identify cFLIP as a major mediator of the TRAIL-resistant SH-SY5Y phenotype and regulator of TRAIL-induced apoptosis in NGF-sensitized TrkA SH-SY5Y cells.

### NGF induces TrkA–cFLIP binding

**Figure 6:**
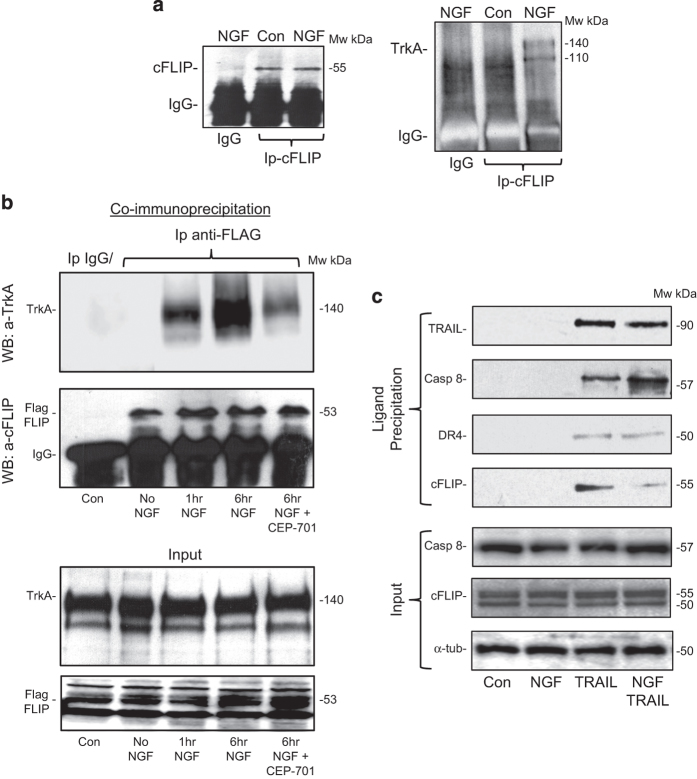
NGF induces TrkA/cFLIP binding and reduces the level of cFLIP at death receptors. (**a**) Representative co-immunoprecipitation western blots demonstrating endogenous cFLIP pull-down of TrkA in immunoprecipitates from total cell extracts (1 mg) of NGF-treated (100 ng/ml for 6 h) but not untreated TrkA SH-SY5Y cells. (**b**) Representative co-immunoprecipitation western blots demonstrating exogenous FLAG-tagged cFLIP pull-down of TrkA by immunoprecipitates in whole cell extracts (300 *μ*g) of NGF-treated (100 ng/ml for 1 and 6 h) but not untreated TrkA SH-SY5Y cells transiently transfected with a FLAG-tagged cFLIP expression vector. Note the reduced levels of TrkA pulled down by exogenous FLAG-tagged cFLIP in TrkA SH-SY5Y cells co-treated with CEP-701 (100 nM) and NGF for 6 h. Input levels of TrkA and cFLIP are also provided (Input). (**c**) Representative western blots demonstrating reduced levels of cFLIP and increased levels of caspase-8 in TRAIL-activated DR4-positive death receptor complexes purified from TrkA SH-SY5Y cells preincubated for 6 h with NGF (100 ng/ml) and then treated for 1 h with biotinylated-TRAIL (500 ng/ml) (NGF TRAIL) as compared with TrkA SH-SY5Y cells preincubated for 6 h in the absence of NGF then treated for 1 h with biotinylated-TRAIL (500 ng/ml) (TRAIL) and TrkA-SH-SY5Y cells treated for 6 h with NGF (100 ng/ml) alone (NGF), and untreated TrkA SH-SY5Y cells (Con). Input levels of caspase-8, cFLIP and *α*-tubulin are provided (Input).

NGF (100 ng/ml for 1–6 h) did not reduce cFLIP mRNA or protein expression in TrkA SH-SY5Y cells nor induce cFLIP degradation (Figure 4a), suggesting an alternative mechanism for sensitization to TRAIL-induced apoptosis. NGF (100 ng for 1 and 6 h), however, induced a binding interaction between TrkA and endogenous cFLIP (Figure 6a) and also between TrkA and exogenous FLAG-tagged cFLIP transiently overexpressed in TrkA SH-SY5Y cells (Figure 6b). The TrkA tyrosine kinase inhibitor CEP-701 (100 nM) reduced the binding interaction between TrkA and FLAG-tagged cFLIP detected following 6 h of incubation with NGF, consistent with a requirement for TrkA tyrosine kinase activity (Figure 6b).

### NGF reduces cFLIP recruitment to activated TRAIL death receptors in TrkA SH-SY5Y cells

The possibility that NGF sensitization of TrkA SH-SY5Y cells to TRAIL-induced death results from cFLIP sequestration was confirmed by comparing cFLIP and caspase-8 levels associated with TRAIL-ligated DR4 death receptor complexes purified from TrkA SH-SY5Y cells preincubated for 6 h in the presence or absence of NGF (100 ng/ml) before 1 h of treatment with biotinylated-TRAIL (500 ng/ml). Ligand-affinity purified, TRAIL-ligated, DR4-positive death receptor complexes from TrkA SH-SY5Y cells that had been pretreated for 6 h with NGF (100 ng/ml) before being treated for 1 h with biotinylated-TRAIL (500 ng/ml) contained lower levels of cFLIP, higher levels of caspase-8 and similar levels of DR4 when compared with complexes purified from TrkA SH-SY5Y cells treated with biotinylated-TRAIL (500 ng/ml), confirming that NGF reduces cFLIP recruitment and increases caspase-8 recruitment to TRAIL-activated death receptors in TrkA SH-SY5Y cells. Similar complexes were not purified from untreated TrkA SH-SY5Y cells or TrkA SH-SY5Y cells treated with NGF (100 ng/ml) alone (Figure 6c).

### NGF sensitization of TrkA SH-SY5Y cells to TRAIL-induced apoptosis is temporary and eventually inhibited by NF-*κ*B and Mcl-1

**Figure 7:**
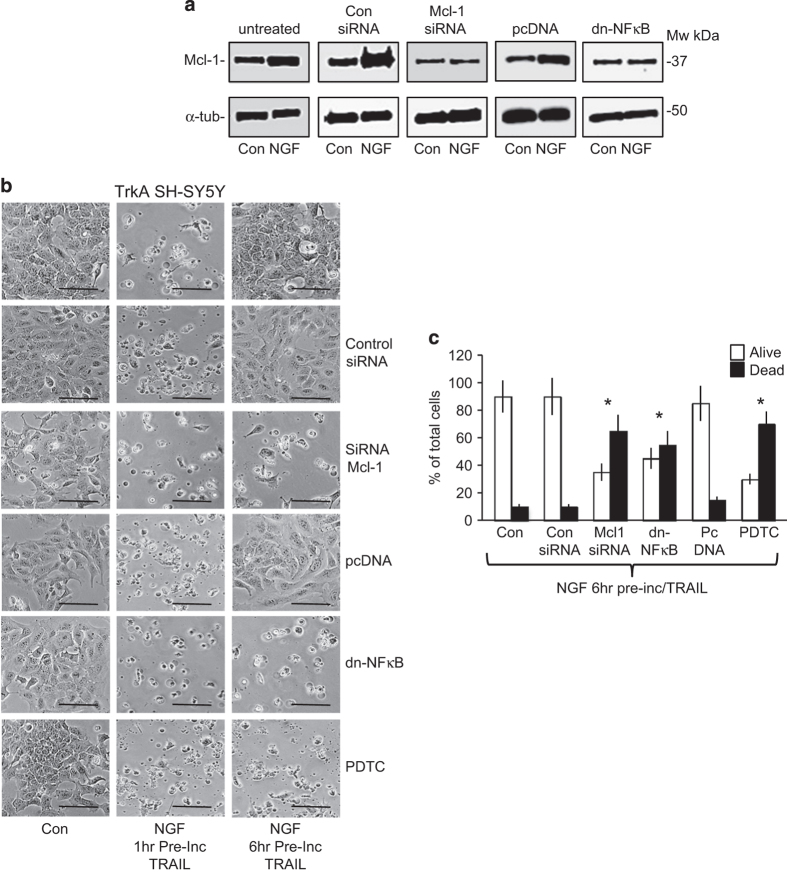
NGF sensitization of TrkA SH-SY5Y cells to TRAIL-induced apoptosis is temporary and regulated by NF-*κ*B and Mcl-1. (**a**) Representative western blots demonstrating NGF (100 ng/ml for 6 h) stimulation of constitutive Mcl-1 expression in whole cell extracts (30 *μ*g) from untreated, control siRNA-treated and transient empty pcDNA-transfected TrkA SH-SY5Y cells, reduced constitutive Mcl-1 expression and loss of NGF-stimulated Mcl-1 expression in total extracts (30 *μ*g) from TrkA SH-SY5Y cells transiently transfected with Mcl-1-specific siRNA duplexes and the loss of NGF-stimulated Mcl-1 expression in cells transiently transfected with dn-NF-*κ*B. (**b**) Representative phase contrast micrographs demonstrating TRAIL-induced apoptosis of TrkA SH-SY5Y cells following 1 h (NGF 1 h pre-Inc TRAIL) but not 6 h of NGF preincubation (NGF 6 h pre-Inc TRAIL) (bar=100 *μ*M), and (**c**) histogram demonstrating TRAIL-induced apoptosis following 6 h of preincubation with NGF in TrkA SH-SY5Y cells transiently transfected with Mcl-1-specific siRNA duplexes, dn-NF-*κ*B expression vector or treated with the NF-*κ*B inhibitor PDTC but not in cells transiently transfected with the empty pcDNA expression vector or nontransiently transfected 6 h NGF pretreated TrkA SH-SY5Y cells (Con). Results are displayed as the mean percentage (%) (±S.D.) alive (white) or dead (black) of total cells in three independent experiments, each performed in duplicate (*significant difference).

In preincubation studies, NGF sensitization of TrkA SH-SY5Y cells to TRAIL-induced apoptosis was time dependent and was detected following preincubation with NGF for 1 h but not 6 h (Figure 7a). This loss of sensitivity to TRAIL-induced apoptosis following 6 h of preincubation with NGF did not associate with loss of TrkA Y674/675 phosphorylation (Figure 4a), considered to be an index of TrkA tyrosine kinase activity,^44^ nor with a reduction in the NGF-induced binding interaction between TrkA and FLAG-tagged cFLIP (Figure 6b). It did associate, however, with a marked increase in the expression of the mitochondrial apoptosis inhibitor Mcl-1,^45^ and to a lesser extent Bcl-xL but not Bcl-2 (Figure 4a). Furthermore, NGF also stimulated the expression of functional DR4 and DR5 but not decoy DcR1 or DcR2 TRAIL receptors, caspase-3, caspase-8, caspase-9, BID or BAX (Bcl-2-associated X protein) (Figures 4a and b, BAX not shown).

The siRNA Mcl-1 knockdown in TrkA SH-SY5Y cells reduced constitutive Mcl-1 expression and inhibited NGF stimulation of Mcl-1 expression (Figure 7a). Mcl-1 knockdown also prevented loss of sensitivity to TRAIL-induced apoptosis, following 6 h of preincubation with NGF (Figures 7b and c). Mcl-1 knockdown, however, did not sensitize NT SH-SY5Y, pcDNA SH-SY5Y or TrkA SH-SY5Y cells to TRAIL-induced apoptosis in the absence of NGF (data not displayed), confirming that Mcl-1 was not directly responsible for the TRAIL-resistant SH-SY5Y phenotype despite being largely responsible for the loss of sensitivity to TRAIL, observed following 6 h of preincubation with NGF.

The transcription factor NF-*κ*B regulates Mcl-1 expression and is activated by NGF in TrkA SH-SY5Y cells.^46–48^ NF-*κ*B involvement in NGF-stimulated Mcl-1 expression and loss of sensitivity to TRAIL following 6 h of NGF preincubation was confirmed by transient expression of dominant-negative NF-*κ*B (dn-NF-*κ*B) and also by incubation with the NF-*κ*B inhibitor pyrrolidine dithiocarbamate (PDTC).^49,50^ Both dn-NF-*κ*B and PDTC reduced NGF stimulation of Mcl-1 expression in TrkA SH-SY5Y cells (Figure 7a) and prevented loss of sensitivity to TRAIL-induced apoptosis in TrkA SH-SY5Y cells, following 6 h of NGF preincubation (Figures 7b and c).

## Discussion

In the present study, we show that NGF sensitizes TrkA-expressing SH-SY5Y cells to TRAIL-induced apoptosis, unveiling a novel pro-apoptotic immunological dimension to NGF interaction with the TrkA receptor in transformed human neuroblasts. This mechanism depends upon TrkA expression, TrkA activation, caspase and BID cleavage and results in type II apoptosis through the intrinsic mitochondrial pathway. We identify cFLIP as the major mediator of the TRAIL-resistant SH-SY5Y phenotype and provide evidence that NGF sensitization to TRAIL-induced apoptosis results from cFLIP sequestration by NGF-activated TrkA. This reduces cFLIP and increases caspase-8 recruitment to TRAIL-activated death receptors, resulting in the cleavage of caspase-8 and BID, in turn leading to OMM permeabilization and type II cell apoptosis through the intrinsic mitochondrial pathway. We identify NF-*κ*B and Mcl-1 as endogenous inhibitors and NF-*κ*B and Mcl-1 inhibitors as exogenous optimizers of this novel NGF/TrkA-mediated TRAIL sensitization response.

The TRAIL-resistant SH-SY5Y phenotype could not be explained by the absence of any single component of the TRAIL-induced apoptotic pathway and, in contrast to some reports,^19,20,27^ our SH-SY5Y cell lines exhibited constitutive functional TRAIL receptors and caspase-8 expression. Furthermore, the relatively low level of decoy (DcR1 and DcR2) relative to functional (DR4 and DR5) TRAIL receptors expression observed suggests that the TRAIL-resistant SH-SY5Y phenotype was not the result of an anti-apoptotic equilibrium between functional and decoy TRAIL receptors. Indirect IF and ligand affinity TRAIL receptor purification confirmed that functional TRAIL receptors were expressed at the cell surface, indicating that differential localization of functional and decoy TRAIL receptors was unlikely to be responsible for the TRAIL-resistant phenotype, as reported previously.^51^ SH-SY5Y cell lines also exhibited low-level expression of the Bcl-2 family proteins, Bcl-2, Bcl-xL and Mcl-1, suggesting that TRAIL resistance was unlikely to depend upon an anti-apoptotic equilibrium between Bcl-2 and BH3-only family proteins.

We identify cFLIP as the major mediator of the TRAIL-resistant SH-SY5Y phenotype, based upon the observations that: TRAIL-resistant cell lines exhibited high-level expression of cFLIP (S and L); TRAIL alone did not induce the cleavage of either caspase-8 or BID consistent with a block at the death receptor level; siRNA cFLIP knockdown sensitized TrkA SH-SY5Y cells to TRAIL-induced apoptosis in the absence of NGF; and transient cFLIP overexpression inhibited TRAIL-induced apoptosis in NGF-sensitized TrkA SH-SY5Y cells. However, NGF did not reduce cFLIP expression or induce cFLIP degradation in TrkA SH-SY5Y cells, implicating an alternative sensitization mechanism. A sensitization mechanism based upon cFLIP sequestration was suggested by NGF stimulation of a CEP-701-sensitive binding interaction between NGF-activated TrkA and cFLIP and was confirmed by a reduction in cFLIP and increase in caspase-8 recruitment to TRAIL-activated death receptor complexes. This adds to a recent report that TrkA binds cFLIP during NGF-induced neuritogenesis,^52^ characterizes TrkA as a TRAIL-activated TRAIL receptor competitor and supports reports that cFLIP competes with caspase-8 to bind TRAIL-activated TRAIL receptors.^53^ This adds a novel dimension to TrkA function as a regulator of cFLIP involvement in death receptor-mediated apoptosis. The sequestration of cFLIP may also extend to MAPKs that also bind cFLIP,^54^ but it remains to be determined whether this may extend to other NT receptors.

NGF sensitization of TrkA SH-SY5Y cells to TRAIL-induced apoptosis was dependent upon TrkA tyrosine kinase activity and was prevented by the TrkA inhibitors CEP-701,^41^ Gö6976,^42^ and GW441756.^43^ Apoptosis was also caspase dependent and prevented by the caspase-3 inhibitor z-VAD-fmk,^55^ the caspase-8 inhibitor z-IETD-fmk^56^ and the caspase-9 inhibitor z-LEHD-fmk.^57^ Bcl-xL overexpression inhibited apoptosis, confirming intrinsic mitochondrial apoptosis pathway involvement that was also supported by the induction of BID cleavage, increased cytosolic levels of the pro-apoptotic mitochondrial proteins cytochrome *C* and OMI/Htr2a and reduced cytosolic levels of the anti-apoptotic protein XIAP, consistent with OMM permeabilization and type II cell apoptotic behaviour.^3,7,58,59^

NGF sensitization of TrkA SH-SY5Y cells to TRAIL-induced apoptosis was temporary and in preincubation studies was detected following preincubation with NGF for 1 h but not 6 h. This time-dependent loss of sensitivity to TRAIL did not associate with the loss of TrkA tyrosine phosphorylation or a reduction in the binding interaction between TrkA and cFLIP. Furthermore, it was also associated with an increase in the expression of functional (DR4 and DR5) over decoy (DcR1 and DcR2) TRAIL receptors, excluding a potential role for an inhibitory equilibrium between functional and decoy TRAIL receptors. Loss of sensitivity did, however, associate with a marked increase in the expression of the mitochondrial apoptosis inhibitors Mcl-1 and to a lesser extent Bcl-xL. Mcl-1 involvement was confirmed by siRNA knockdown that not only prevented NGF stimulation of Mcl-1 expression but also prevented the loss of TrkA SH-SY5Y cell sensitivity to TRAIL-induced apoptosis following 6 h of preincubation with NGF. In contrast to its effects upon TRAIL receptor, Mcl-1 and Bcl-xL expression, NGF did not alter the expression of caspases, Bcl-2, BID or BAX.

The transcription factor NF-*κ*B regulates Mcl-1 expression^46,47^ and is activated by NGF in TrkA SH-SY5Y cells.^48^ A role for NF-*κ*B in the regulation of sensitivity to TRAIL was also confirmed using the NF-*κ*B inhibitors dn-NF-*κ*B and PDTC,^49,50^ both of which reduced NGF stimulation of Mcl-1 expression and prevented the loss of TrkA SH-SY5Y cell sensitivity to TRAIL-induced apoptosis following 6 h of preincubation with NGF. Interestingly, NGF stimulated Mcl-1 and Bcl-xL but not Bcl-2 expression in TrkA SH-SY5Y cells. Although all three genes have been reported to be NF-*κ*B-regulated,^46,47,60,61^ Bcl-2 is differentially regulated by the transcription factor CREB (cAMP response element-binding protein)^62^ that is inhibited in SH-SY5Y cells.^63^ We are further investigating this differential regulation of Mcl-1, Bcl-xL and Bcl-2.

The combination of NGF plus TRAIL inhibited TrkA SH-SY5Y tumourigenicity by >95%, more than expected, considering that NGF plus TRAIL induced no more than 70% cell death in a temporary sensitization effect. This can be explained by the differentiation promoting effects of NGF (see this study, and Poluha *et al.* ^64^ and Tacconelli *et al.* ^65^) that, despite not inducing apoptosis, caused 50% inhibition of TrkA SH-SY5Y tumourigenicity in the absence of TRAIL.

In conclusion, we report a novel important pro-apoptotic immunological dimension to the interaction between NGF and the TrkA receptor in sensitizing neuroblastoma cells to TRAIL-induced apoptosis. We propose that this mechanism depends upon cFLIP sequestration by activated TrkA receptors and characterizes TrkA as a novel competitive regulator of TRAIL-induced apoptosis that competes with death receptor complexes for cFLIP. Our observations may help to explain the association between TrkA expression, better prognosis and spontaneous remission in NB^1^ and provide a novel potential pro-apoptotic therapeutic strategy, to be developed in the future, for the use of ‘painless’ forms of NGF^66^ or alternative TrkA agonists, together with TRAIL and inhibitors of NF-*κ*B and/or Mcl-1 in favourable and unfavourable NBs that express TrkA and exhibit cFLIP-mediated TRAIL resistance. This novel approach may work best as a first-line therapy, as TRAIL receptor activation has recently been reported to promote mutated ki-Ras-driven metastatic progression^67^ and ki-Ras mutations are detected in relapsed but not primary NBs.^68–70^

## Materials and methods

NT, pcDNA control-transfected and TrkA-transfected SH-SY5Y NB cell lines have been described previously.^65^ Cells were grown in RPMI, supplemented with appropriate antibiotics (Zeocin for stable transfectants, penicillin and streptomycin) and 10% fetal calf serum. TrkA inhibitors Gö6976^42^ and GW441756;^43^ the NF-*κ*B inhibitor PDTC;^50^ the cell-permeable caspase-3 inhibitor z-VAD-fmk, caspase-8 inhibitor z-IETD-fmk and caspase-9 inhibitor z-LEHD-fmk; NGF and human recombinant TRAIL were purchased from Sigma-Aldrich (St. Louis, MO, USA). The pan Trk inhibitor, CEP-701,^41^ was kindly supplied by Cephalon Inc. (West Chester, PA, USA). Antibodies against TrkA, *α*-tubulin, cFLIP, caspase-8, caspase-9, BID and Mcl-1 were purchased from Santa Cruz (Santa Cruz, CA, USA). Antibodies against phosphorylated Y674/675 TrkA and Caspase-3 were from Cell Signalling (Danvers, MA, USA). Antibodies against DR4, DR5, DcR1 and DcR2 were from ANASPEC (Seraing, Belgium). Antibodies against Bcl-2 and Bcl-xL were from Abcam (Cambridge, UK). Hybond C-extra nitrocellulose membranes and ECL solutions were purchased from Amersham International (Bedford, UK). RNA-easy RNA purification kits were purchased from Qiagen (Hilden, Germany). Mammalian pcDNA expression vectors for Bcl-xL and Flag-tagged cFLIP were kindly provided by Dr. Francesca Zazzeroni (University of L’Aquila, L’Aquila, Italy) and have been described previously.^71^ The dn-NF-*κ*B expression vector has been previously described.^49^

### Transient overexpression of Bcl-xL, cFLIP and dn-NF-*κ*B

Bcl-xL, flag-tagged cFLIP and dn-NF-*κ*B were overexpressed in SH-SY5Y cell lines by transient transfection of mammalian expression vectors bearing Bcl-xL, FLAG-tagged cFLIP^71^ or dn-NF-*κ*B.^49^ Briefly, 0.6 *μ*g/ml of each vector was transfected into subconfluent cell cultures using FUGENE transfection reagent as directed by the manufacturer (Promega, Madison, WI, USA). At 6 h of transfection, medium was replaced with fresh growth medium (RPMI/10% FCS/glutamine plus antibiotic) and the cells were left for 48 h, at which time they were utilized for experimentation. Overexpression of Bcl-xL and Flag-tagged cFLIP was verified by western blotting of total cell extracts (20 *μ*g).

### SiRNA cFLIP and Mcl-1 knockdown

The cFLIP and Mcl-1 expression was knocked down in NB cells using a TriFECTa Dicer-Substrate RNAi kit, employing three cFLIP-specific or three Mcl-1-specific Dicer-Substrate siRNA duplexes, as described by the manufacturer (Integrated DNA Technologies, Coralville, IA, USA). Briefly, cells were subjected to 48 h of transient transfection with either 25 nM negative control siRNA duplex (provided with the kit) or 25 nM of a mix of cFLIP-specific or Mcl-1-specific siRNA duplexes, using INTERFERin *in vitro* siRNA transfection reagent, as described by the manufacturer (Polyplus Transfection Inc., New York, NY, USA). Sham-transfected controls received transfection reagent alone. The siRNA knockdown of cFLIP and Mcl-1 expression was confirmed by western blot comparison with *α*-tubulin in whole cell extracts (20 *μ*g).

Mcl-1 siRNA sets were as follows: 5′-AGCCUAGUAUGUCAAUAAAGCAAAT-3′ and 5′-AUUUGCUUUUAUUGACAUACUA GGCUUA-3′; 5′-GGAACAAAUCUGAUAACUAUGCAGG-3′ and 5′- CCUGCAUAGUUAUCAGAUUUGUUCCAC-3′; and 5′-CAAGUGCAUAGAUGUGAAUUGGUTT-3′ and 5′-AAACCAAUUCACAUCUAUGCACUUGUU-3′. cFLIP siRNA sets were as follows: 5′-UGAGUUGGAGAAACUAAAUCUGGTT-3′ and 5′-AACCAGAUUUAGUUUCUCCAACUCAAC-3′; 5′-CGAAGACCCUUGUGAGCUUCCCUAG-3′ and 5′-CUAGGGAAGCUCACAAGGGUCUUGCAG-3′; and 5′-GCCGAGGCAAGAUAAGCAAGGAGAA-3′ and 5′-UUCUCCUUGCUUAUCUUGCCUCGGCCC-3′.

Transfection efficiency was confirmed using a HPRT-S1 DS positive control and validated using a negative control duplex (NC1) not present in the human genome, as described by the manufacturer (Integrated DNA Technologies; www.IDTDNA.com).

### Cell extraction, immunoprecipitation and western blotting

Cells were extracted in lysis buffer (PBS containing 0.5% sodium deoxycholate, 1% NP-40, 0.1% SDS, 1 mM sodium orthovanadate, 1 mM PMSF, 1 *μ*g/ml of pepstatin A and aprotinin) and protein concentrations were calculated by Bradford protein concentration assay (Sigma-Aldrich). Cytoplasmic levels of cytochrome *C* and Omi/Htr2a were assessed in the same lysates, following removal of mitochondria. Briefly, cell lysates were centrifuged at 3 000×*g* for 5 min at 4 °C, supernatants containing cytosol and mitochondria recovered and centrifuged at 15 000×*g* for 30 min at 4 °C. The mitochondria-free supernatant was taken and recentrifuged at 15 000×*g* at 4 °C to give the mitochondria-free sample for western blotting. Samples were mixed with reducing sodium dodecyl sulphate-polyacrylamide gel electrophoresis (SDS-PAGE) sample buffer and subjected to reducing SDS-PAGE/western blotting. Briefly, proteins separated by reducing SDS-PAGE were transblotted onto Hybond C+ nitrocellulose membranes by electrophoresis (Amersham International) and the membranes subsequently air-dried. Nonspecific protein binding site on membranes were blocked by incubation for 2 h in 5% non-fat milk in Tris-buffered saline and Tween-20 (TBS-T) before incubation with primary antibodies, at recommended dilutions, for 2–16 h at 4 °C. Membranes were then washed in TBS-T, incubated with secondary HRP-conjugated antibodies (Jackson ImmunoResearch Laboratories, West Grove, PA, USA) diluted in blocking solution and immunoreactive species detected by chemiluminescence reaction, as directed by the manufacturer (Amersham International).

### RNA purification and reverse transcription-PCR (RT-PCR)

RT reactions were performed on total RNAs (1 *μ*g), purified using RNA-easy Plus, as described by the manufacturer (Qiagen) using the Moloney Murine Leukemia virus RT kit as detailed by the manufacturer (LifeTechnologies, Inc., Paisley, UK). RT reactions were subjected to PCR using the following primers: GAP: 5′-AGGTCCACCACTGACAGTT-3′ (forward) and 5′-CTGCACCACCAACTGCTTAG-3′ (reverse) (300 bp); TrkA: 5′-AGAAGCTGCAGTGTCATGGG-3′ (forward) and 5′-ATTGAGCACGGAGCCATTGA-3′ (reverse) (452 bp); DcR1: 5′-GAAGAATTTGGTGCCAATGCCACTG-3′ (forward) and 5′-CTCTTGGACTTGGCTGGGAGATGTG-3′ (reverse) (612 bp); DcR2: 5′-CCCCCGGCAGGACGAAGTT-3′ (forward) and 5′-CTCCTCCGCTGCTGGGGTTTT-3′ (reverse) (418 bp); DR4: 5′-ACTTTGGTTGTTCCGTTGCTGTTG-3′ (forward) and 5′-GGCTTTCCATTTGCTGCTCA-3′ (reverse) (214 bp); DR5: 5′-CTGAAAGGCATCTGCTCAGGTG-3′ (forward) and 5′-CAGAGTCTGCATTACCTTCTAG-3′ (reverse) (347 bp); cFLIPs: 5′-GGACCTTGTGGTTGAGTTGG-3′ (forward) and 5′-ATCAGGACAATGGGCATAGG-3′ (reverse) (241 bp); cFLIP_L_: 5′-GGCTCCCAGAGTGTGTATGG-3′ (forward) and 5′-AGCTTCTCGGTGAACTGTGC-3′ (reverse) (249 bp); Bcl-2: 5′-GACTTCGCCGAGATGTCC-3′ (forward) and 5′-CAAGCTCCCACCAGGGCCAAAC-3′ (reverse) (356 bp); Bcl-xL: 5′-GTGAATTCTGAGGCCAAGGGAAC-3′ (forward) and 5′-GAACGGCGGCTGGGATACTTT TG-3′ (reverse) (373 bp); caspase-8: 5′-TCTGGAGCATCTGCTGTCTG-3′ (forward) and 5′-CCTGCCTGGTGTCTGAAGTT-3′ (reverse) (427 bp); caspase-10: 5′-GGGAACGGACACACAACTCT-3′ (forward) and 5′-CTAGCTTTTGGCCCTGACTG-3′ (reverse) (293 bp); caspase-3: 5′-TTAATAAAGGTATCCATGGAGAACACT-3′ (forward) and 5′-TTAGTGATAAAAATAGAGTTCTTTTGTGAG-3′ (reverse) (849 bp); BAX: 5′-AAGAAGCTGAGCGAGTGT-3′ (forward) and 5′-GGAGGAAGTCCAATGTC-3′ (reverse) (256 bp); Mcl-1: 5′-AAGCCAATGGGCAGGTCT-3′ (forward) and 5′-TGTCCAGTTTCCGAAGCAT-3′ (reverse) (121 bp).

For each primer set, PCRs were performed on reverse transcription reactions serially diluted from 1 to 1 : 1000. Reactions below saturation were compared by densitometric analysis of Jpeg images of ethidium bromide stained gels, using ImageJ64 software.^72^

### Analysis of apoptosis and the cell death assay

TRAIL-induced apoptosis was confirmed by *in situ* Tunel assay as described by the manufacturer (R&D Systems, Minneapolis, MN, USA). For DNA ladder analysis, cells were collected by centrifugation at 5000 r.p.m. for 5 min, lysed, incubated at room temperature for 10 min and then incubated for 5 min at 65 °C. Samples, cooled to room temperature for 5 min, were mixed with 700 *μ*l of chloroform–isoamyl alcohol and centrifuged for 5 min at 12 000 r.p.m. The aquatic (upper) phase was transferred, an equal volume of cold isopropanol was added, mixed gently by inversion and centrifuged for 5 min at 12 000 r.p.m. The supernatant was discarded and pellet air-dried for 30 min. Dried DNA was dissolved in 50 *μ*l of distilled water, quantified in a spectrophotometer (NanoDrop 1000, Wilmington, DE, USA) and analysed by 1.5% agarose gel electrophoresis.

Cell death was routinely assayed using a modification of previously described methods.^73,74^ Briefly, cells were washed once in Ca^2+^-free PBS, detached with ice-cold PBS containing 1 mM EDTA, transferred to sterile 15 ml tubes, centrifuged for 5 min at 1000×*g* at 4 °C, washed with ice-cold PBS and repelleted by centrifugation at 1000 ×*g* for 5 min at 4 °C. Cell pellets were resuspended in 25 *μ*l of PBS containing 2 *μ*l of acridine orange/ethidium bromide solution (100 μg/ml acridine orange and 100 *μ*g/ml ethidium bromide in PBS) plated onto glass slides and examined immediately under a Zeiss (Carl Zeiss, Oberkochen, Germany) ‘Axioplan-2’ fluorescence microscope. Representative fields were digitally photographed under identical exposure conditions and the number of dead cells (orange/red nuclei) and live cells (green nuclei) counted. In addition to this assay, phase contrast micrographs of parallel cultures were used to confirm changes in the relative percentage of adherent and suspension (predominantly apoptotic) cells following TRAIL treatment.

### Tumour growth in soft agar

For substrate-independent tumour growth assays, 1×10^4^ cells in single-cell suspension (passed through a gauge × 18 syringe needle) were mixed in a 0.3% solution of agar (BiTech Difco-BD, Milan, Italy) in RPMI containing 5% FCS at 37 °C, with or without NGF (100 ng/ml) and/or TRAIL (200 ng/ml) and layered onto a solid 0.6% agarose substrate also with and without NGF and/or TRAIL, prepared in the same growth medium. Following top phase agar solidification, complete medium was added and replaced every 2 days. Tumour spheroid growth was monitored over a 14-day period by phase contrast microscopy. Tumour spheroids were counted in 10 random fields at ×10 magnification.

### Isolation of TRAIL-activated TRAIL receptor complexes

TRAIL-ligated death receptor complexes were purified from TrkA SH-SY5Y cells by ligand affinity precipitation, as previously described.^75^ Briefly, biotinylated TRAIL was prepared by incubating TRAIL (1 mg/ml) with Sulfo-NHS-LC-Biotin (1mg/ml; Thermo Fisher Scientific, Waltham, MA, USA) for 1 h on ice. The reaction was stopped by adding 1 : 10 volume of 1 M Tris-HCl (pH 7.5) and unincorporated biotin removed by buffer exchange into 150 mM NaCl, 30 mM HEPES (pH 7.5) using PD-10 columns (Amersham Pharmacia Biotech, Piscataway, NJ, USA). For ligand affinity precipitation, cells (5×10^6^ cells per sample) were washed twice in RPMI at 37 °C and incubated with 1 *μ*g/ml biotinylated TRAIL for 1 h. Death receptor complex formation was stopped by the addition of 15 volumes of ice-cold PBS and cells were then lysed in 4.5 ml of lysis buffer (30 mM TRIS-HCl (pH 7.5), 150 mM NaCl, 10% glycerol, 1% Triton X-100, supplemented with complete protease inhibitor cocktail; Roche Diagnostics, Mannheim, Germany). TRAIL receptor protein complexes were precipitated from lysates by co-incubation with 20 *μ*l of Streptavidin beads (Pierce) for 3 h at 4 °C with rotation. Ligand affinity precipitates were washed four times in lysis buffer, eluted from beads in reducing SDS-PAGE sample buffer and subjected to SDS-PAGE western blotting.

### Statistical analysis

Data were analysed statistically by Student’s *t*-test and statistical significance was associated with probabilities of ⩽0.05.

## Abbreviations

NB: neuroblastoma
TNF: tumour necrosis factor
TRAIL: TNF-related apoptotis-inducing ligand
cFLIP: cellular FLICE-like inhibitory protein
NF-*κ*B: nuclear factor-*κ* binding
dn-NF-*κ*B: dominant-negative NF-*κ*B
NGF: nerve growth factor
Trk: tropomyosin-related kinase
Mcl-1: myeloid cell leukaemia-1
Bcl-2: B-cell lymphoma 2
Bcl-xL: B-cell lymphoma extra large
BH3-only: Bcl-2 homology domain 3-only
BID: BH3 interacting domain death agonist
FADD: Fas-associated protein with death domain
OMM: outer mitochondrial membrane
DcR: decoy receptor
siRNA: small interfering RNA
XIAP: X-linked inhibitor of apoptosis
AIF: apoptosis-inducing factor
BAX: Bcl-2-associated X protein
PDTC: pyrrolidine dithiocarbamate
NT: nontransfected
CREB: cAMP response element-binding protein
TBS-T: Tris-buffered saline and Tween-20
SDS-PAGE: sodium dodecyl sulphate-polyacrylamide gel electrophoresis
RT-PCR: reverse transcription-PCR
SMAC/DIABLO: second mitochondria-derived activator of caspases.
